# Platelet gene signatures detecting pulmonary artery stenosis in patients with pulmonary hypertension

**DOI:** 10.1186/s13023-026-04307-4

**Published:** 2026-03-13

**Authors:** Junhao Jin, Hongling Su, Zunmin Wan, Yating Zhao, Hongfan Zhao, Aiping Tang, Ya Ma, Huan Liu, Tongtong Gao, Like Ma, Aqian Wang, Bo Li, Kaiyu Jiang, Fu Zhang, Yunhe Zhang, Mei Jiang, Chenxi Zhang, Min Zhang, Yunshan Cao

**Affiliations:** 1 Heart, Lung and Vessels Center, Sichuan Provincial People’s Hospital, University of Electronic Science and Technology of China, Chengdu, China; 2 The First School of Clinical Medicine, Lanzhou University, Lanzhou, China; 3 Department of Cardiology, Pulmonary Vascular Disease Center, Gansu Provincial Hospital, Lanzhou, China; 4 Sichuan Provincial Key Laboratory for Human Disease Gene Study, Genome Sequencing Center, Department of Laboratory Medicine, Sichuan Provincial People’s Hospital, School of Medicine, University of Electronic Science and Technology of China, Chengdu, China; 5 School of Medicine, Jiangsu University, Zhenjiang, China; 6 The First Clinical Medical School, Gansu University of Chinese Medicine, Lanzhou, China; 7 Central Laboratory, Nanjing Chest Hospital, Affiliated Nanjing Brain Hospital of Nanjing Medical University, Nanjing, China; 8 Clinical Research Center, Sichuan Provincial People’s Hospital, University of Electronic Science and Technology of China, Chengdu, China

**Keywords:** Pulmonary hypertension, Pulmonary artery stenosis, Biomarkers, Blood platelets

## Abstract

**Background:**

Pulmonary artery stenosis (PAS) is a major cause of pulmonary hypertension (PH). The advancement of non-invasive biomarkers to identify PAS in high-risk individuals has the potential to enhance the precision of clinical evaluations related to PH. This study aimed to present evidence that gene expression data within blood platelets could be valuable for detecting PAS in patients with PH.

**Methods:**

Platelets were isolated from 241 PH patients and 98 healthy controls for RNA sequencing. Differentially expressed genes (DEGs) were identified between PAS and non-PAS, and between chronic thromboembolic pulmonary hypertension (CTEPH) and PH caused by fibrosing mediastinitis (FM-PH). Three machine learning algorithms—random forest (RF), extreme gradient boosting (XGBoost), and Boruta—were applied to select platelet genes of discriminative capability. Genes identified by all three algorithms were used for subsequent model construction. Fourteen predictive models were trained and validated using repeated fivefold cross-validation. Functional enrichment and gene set enrichment analyses (GSEA) were performed.

**Results:**

Compared with the non-PAS group, PH patients with PAS exhibited 244 upregulated and 1,051 downregulated genes in platelets. GSEA revealed upregulation of pathways including platelet activation, fluid shear stress and atherosclerosis, and Rap1 signaling, alongside downregulation of PI3K-Akt and mTOR signaling in patients with PAS. Six platelet RNAs were identified by RF, XGBoost, and Boruta for differentiating PAS from non-PAS. The RF model, with NOTCH1 contributing most significantly (highest mean decrease in Gini index), achieved the highest area under the curves (AUCs) of 0.946, 0.862, and 0.749 in the training, internal, and external validation sets, respectively. Within PAS, 669 genes were upregulated and 697 downregulated in CTEPH versus FM-PH. Pathways including vascular smooth muscle contraction and blood vessel remodeling were positively enriched in platelets from patients with CTEPH compared to FM-PH. Two genes, PPP1CA and MAPRE1, were shared across all three feature selection algorithms for discriminating between CTEPH and FM-PH. The RF model based on these genes achieved the highest AUCs of 0.960, 0.925, and 0.961 across the training, internal, and external validation sets.

**Conclusions:**

Platelet-derived biomarkers are potentially useful in identifying PAS and differentiating its subtypes in individuals with PH.

**Supplementary Information:**

The online version contains supplementary material available at 10.1186/s13023-026-04307-4.

## Background

Pulmonary hypertension (PH) is a progressive disease encompassing a heterogeneous group of disorders with the common feature of elevated pulmonary artery pressures [1]. The World Health Organization etiologically categorizes PH into 5 clinical groups and defines PH according to hemodynamic parameters [2].

Pulmonary artery stenosis (PAS) refers to either a complete blockage or a partial narrowing of the cross-sectional area of the pulmonary artery, which can occur due to several factors such as chronic thromboembolic pulmonary hypertension (CTEPH), Takayasu arteritis, peripheral PAS (PPS), congenital PAS, and fibrosing mediastinitis (FM) [3]. In the absence of appropriate treatment, patients with chronic PAS may develop PH owing to the alteration of normal hemodynamics in the pulmonary circulation [4, 5]. A series of studies have demonstrated the essential role of surgery or interventional therapy in treating patients with PH caused by chronic PAS [6–8], and early intervention could result in favorable outcomes in these patients [9, 10]. Compared with chronic PAS, PH without PAS (non-PAS) is mainly treated with drug therapies such as targeted therapies for pulmonary arterial hypertension (PAH). Therefore, the identification of PAS in evaluating patients with PH is crucial.

Delays in the diagnosis of PAS [11, 12] could pose challenges to the management of PH caused by PAS. Computed tomography pulmonary angiography (CTPA) is commonly used to identify PAS; however, it requires contrast agents and involves exposure to radiation. Additionally, it may not be accessible to certain patients owing to hypersensitivity reactions, renal insufficiency, or pregnancy. Furthermore, many clinicians encounter challenges in differentiating PH caused by FM (FM-PH) from CTEPH on CTPA. Consequently, advancing non-invasive biomarkers for the early identification of PH caused by PAS in high-risk individuals has the potential to enhance the precision of clinical evaluations of PH and to positively impact the overall prognosis and quality of life of patients.

The lung is one of the sites for platelet biogenesis [13–16], and several diseases such as lung cancer [17] and inflammatory disorders [18] are known to alter platelet function. Inflammation and proliferation within the blood vessel wall have been reported to mediate chronic PAS and PH [19, 20], and platelets play a role in vascular inflammation [21], which indicates the potential association between pathogenesis of PAS and platelet function. At present, PAS in clinical practice arises from a variety of etiologies. Whether alterations in platelet function serve as a triggering factor in the development of PAS, or instead occur secondary to PAS-induced hemodynamic changes, remains unclear and warrants further investigation.

Recent findings indicated that platelet RNAs could serve as biomarkers for diagnosing PH [22] and differentiating between pre- and post-capillary PH [23]. However, data regarding platelet-based biomarkers for discriminating between PAS and non-PAS in patients with PH remain scarce. This study aimed to analyze the transcriptional characteristics of blood platelets from patients with PH and hypothesized that RNA signatures based on blood platelets could discriminate between PH caused by PAS and non-PAS and identify distinct subtypes of PAS that cause PH (CTEPH and FM-PH).

## Methods

### Study population

This study complied with the ethical guidelines of Helsinki, as reflected in the approval of the ethics regulator at Gansu Provincial Hospital (no. 2024-826). Patients who visited Gansu Provincial Hospital for diagnostic right heart catheterization (RHC) or treatment for PAS between February 2022 and September 2023 were prospectively enrolled. Informed consent was obtained from all the patients. Healthy subjects who were verbally queried not to have a medical history of pulmonary hypertension, as well as for information regarding smoking status, medication use, and comorbidity, were enrolled as control subjects, and all donors provided informed consent. The diagnoses of PAH, PH due to lung diseases (Group 3 PH), CTEPH, and FM-PH were made according to the current guideline of PH [1] and imaging characteristics of FM [24]. Patients with PAS included CTEPH and FM-PH, whereas patients without PAS included PAH and Group 3 PH.

### RHC and echocardiographic examination

During RHC, hemodynamic profile was evaluated, including measurements of mixed venous oxygen saturation (SvO_2_), right atrial pressure (RAP), mean pulmonary arterial pressure (mPAP), pulmonary artery wedge pressure (PAWP), and pulmonary vascular resistance (PVR). Cardiac output (CO) was determined using either the Fick method or thermodilution method, and the cardiac index (CI) was obtained by dividing the CO by the body surface area. Experienced ultrasonologists performed the transthoracic echocardiography in accordance with the latest recommendations [25].

### Blood draw, platelet isolation, and platelet RNA-sequencing (RNA-seq)

Whole venous blood from patients diagnosed with PH and healthy control subjects was collected from the internal jugular vein during RHC or a puncture from the antecubital vein. Blood samples were collected in two 10 mL EDTA-coated purple-capped BD Vacutainers. The platelet isolation kit (PLTMaster™, China) was used for platelet isolation. Samples were processed following standard protocols for platelet isolation, using 2-step centrifugation process and membrane filtering within 24 h after blood collection. All samples were stored frozen at -80 ℃ after platelet isolation. Venous blood samples for laboratory measurements of platelet counts, platelet distribution width (PDW), plateletcrit (PCT), and mean platelet volume (MPV) were also obtained, collected in citrated tubes containing EDTA potassium salt, and then transferred to the hospital laboratory within 30 min.

All platelet samples were initially purified using RNA purification magnetic beads (Vazyme, #N412), followed by RNA processing using a direct-lysis and full-length cDNA amplification strategy with the Discover-sc WTA Kit V2 (Vazyme, #N711). This protocol employed oligo(dT)-primed reverse transcription without an independent rRNA depletion step, thereby selectively capturing polyadenylated mRNA for downstream sequencing. Library construction was performed using TruePrep DNA Library Prep Kit V2 for Illumina (Vazyme, #TD503) with dual indexing. RNA amplification and library construction were performed by the same technician to reduce confounding effects. The average size and quality of each cDNA library were assayed using an Agilent Bioanalyzer 2100, and concentrations were determined by Qubit for proper dilution and equilibration. Indexed libraries (40 samples per group, nine groups in total) were pooled and sequenced on an Illumina NovaSeq 6000 platform using the S4 Reagent Kit v1, generating 150-bp paired-end reads (2 × 150 bp) with a target depth of approximately 45 million reads per sample. The output binary base call (BCL) files were converted to FASTQ format (a text-based format for storing nucleotide sequences and their corresponding quality scores) and demultiplexed. Clean reads were obtained by removing adapters, poly-N sequences, and low-quality reads. Quality control metrics including sequencing depth, clean Q30, and mapped ratio of total reads and mRNA were calculated.

### Platelet transcriptome analysis

Cutadapt software (Version: 4.1) [26] was used to remove adapter sequences from high-throughput sequencing reads, and the processed reads were aligned against the reference human transcriptome GRCh38/hg38 using HISAT2 software (Version: 2.2.1) [27]. Gene-level counts were quantified using featureCounts software (Version: 2.0.3) [28]. Genes were considered to be expressed if they had at least 10 counts in at least two samples. Gene expression data were normalized to transcripts per million (TPM) and log_2_-transformed. Principal component analysis (PCA) was used for dimension reduction of the expression matrix. The R package limma (Version: 3.62.2) [29] was used to remove the batch effect of patient age using the “removeBatchEffect” function and to identify differentially expressed genes (DEGs) using samples from the training set. The Benjamini-Hochberg method for controlling the false discovery rate (FDR) was employed for multiple comparisons. Significant variance in expressed transcripts was pre-specified as transcripts with an FDR < 0.05 and |log_2_fold change| > 0.58.

Enrichment analysis and gene set enrichment analysis (GSEA) [30] using the Kyoto encyclopedia of genes and genomes (KEGG) [31] gene sets were performed via the R packages clusterProfiler (Version: 4.6.2) [32] and DOSE (Version: 2.3.5) [33]. The enrichment score (ES) was calculated by walking down the ranked gene list and increasing or decreasing a running-sum statistic according to the presence of genes in the predefined gene set. The ES was then normalized (NES) by dividing it by the mean ES obtained from 1,000 random permutations of the gene labels. A *p* value < 0.05 was considered statistically significant.

xCell [34] was used to generate enrichment scores for megakaryocytes and platelets based on the RNA-seq data.

### Screening and validation of markers identifying PAS and its subtype

Patients with PH from Gansu province were randomly allocated to the training and internal validation sets (70/30 training-internal validation split), whereas patients residing outside Gansu province constituted the external validation set. To identify robust diagnostic biomarkers, three machine learning algorithms—Boruta [35], random forest (RF) [36], and extreme gradient boosting (XGBoost) [37]—were applied using the R packages Boruta (Version: 8.0.0) [38], randomForest (Version: 4.7.1.2) [39], and xgboost (Version: 1.7.11.1) [40]. For the Boruta algorithm, feature selection was conducted using a wrapper approach built on RF classifiers. Genes classified as “Confirmed” by the algorithm were retained as statistically significant predictors. The RF model was constructed using 500 trees and a randomly selected subset of variables (mtry optimized by grid search). Feature importance was ranked according to the Mean Decrease Gini index, and genes with a value > 0.5 were considered highly informative. For XGBoost, a gradient boosting classifier (objective = binary: logistic, eta = 0.1, max_depth = 4) was trained. Feature importance was evaluated by the gain metric, representing each variable’s contribution to performance improvement. Genes with a gain > 0 were defined as relevant predictors. The intersection of genes identified by all three algorithms was used for subsequent model construction to ensure stability and robustness.

By only using the platelet genes that were commonly selected by all three feature selection methods, 14 machine learning classifiers including RF, XGBoost, LightGBM, CatBoost, support vector machine (SVM), logistic regression (LR), k-nearest neighbor (KNN), Naïve Bayes (NB), decision tree (DT), neural network (NNET), linear discriminant analysis (LDA), elastic net (ENET), and gradient boosting machine (GBM) were implemented. Each model was trained using fivefold cross-validation (CV) repeated five times with class probability estimation. Hyperparameters were tuned via grid search, and training was performed under a parallel computing environment to optimize efficiency. Model performance was evaluated during training and validation using multiple criteria: accuracy, sensitivity, specificity, receiver operating characteristic (ROC) curve, area under the curve (AUC), and F1-score. From the confusion matrix, we derived the following evaluation metrics: sensitivity (true positive (TP) rate) = TP/(TP+ false negative (FN)), specificity (true negative (TN) rate) = TN/(TN+ false positive (FP)), accuracy = (TP + TN)/(TP + FP+TN + FN), positive predictive value (PPV) = TP/(TP + FP), negative predictive value (NPV) = TN/(TN + FN), and the F1-score = 2TP/(2TP + FP+FN).

### Statistical analysis

Categorical variables were represented as proportions (%), while continuous variables were described as mean (standard deviation, SD) or median (interquartile range, IQR), as appropriate. To compare the differences across groups, one-way analyses of variance (normal distribution), Kruskal-Wallis tests (skewed distribution), and chi-square tests (categorical variables) were undertaken. All statistical analyses were carried out by R software (Version 4.1.2). By a two-tailed testing, a *p* value < 0.05 was declared statistically significant.

## Results

### Baseline characteristics of study population

The baseline characteristics of the study population were presented in Table 1. Blood platelets were obtained from patients with PAH (*n* = 75), Group 3 PH (*n* = 19), CTEPH (*n* = 25), and FM-PH (*n* = 122), as well as from healthy controls (*n* = 98). Patients diagnosed with PH had higher age than controls (PH, 57.7 ± 15.2 years; controls, 53.2 ± 14.9 years). Laboratory test results regarding platelet (platelet counts, MPV, PDW, and PCT) were not significantly different between PH and control groups. The PAS group demonstrated elevated platelet counts and PDW relative to the non-PAS group along with reduced MPV and PCT levels. Clinical data indicated a markedly reduced 6-min walk distance (6MWD), serum N-terminal pro-B-type natriuretic peptide (NT-proBNP) levels, right atrium area, and right ventricular end-diastolic area in patients with PAS. Patients with PAS were more likely to have comorbidities such as chronic obstructive pulmonary disease (COPD), hypertension, hyperlipidemia and type 2 diabetes.

**Table 1:** Clinical characteristics of study population

	Healthy control *N* = 98	PH *N* = 241	Non-PAS *N* = 94	PAS *N* = 147
Age, years	53.2 (14.9)	57.7 (15.2) ^$^	49.5 (31.2, 58.8)	66.0 (59.0, 71.0) ^***^
Female, %	66 (67.3%)	116 (48.5%) ^$^	64 (68.1%)	54 (36.7%) ^***^
PLT, 10^9^/L	216 (172, 245)	204 (162, 255)	182 (146, 222)	219 (169, 268) ^***^
PCT, %	10.0 (10.0, 11.0)	10.0 (10.0, 11.0)	11.0 (10.0, 12.0)	10.0 (10.0, 11.0) ^**^
PDW, fL	0.22 (0.18, 0.25)	0.21 (0.18, 0.26)	0.20 (0.16, 0.23)	0.22 (0.18, 0.27) ^**^
MPV, fL	12.0 (10.0, 14.0)	12.0 (10.0, 13.0)	13.0 (11.0, 15.0)	12.0 (10.0,13.0) ^***^
WHO-FC				
I-II	/		49 (52.1%)	73 (49.7%)
III-IV	/		45 (47.9%)	74 (50.3%)
6MWD, meter	/		379 (296, 425)	330 (250, 387) ^**^
NT-proBNP, pg·mL^− 1^	/		736 (120, 1787)	252 (112, 693) ^**^
Right atrium area, mm^2^	/		19.0 (16.0, 27.0)	16.0 (14.0, 20.0) ^***^
RV end-diastolic area, mm^2^	/		29.5 (22.2, 35.8)	22.6 (17.0, 28.0) ^***^
LVEF, %	/		68.0 (63.0, 72.0)	68.0 (65.0, 71.0)
TAPSE, mm	/		20.0 (17.0, 23.0)	20.0 (18.0, 22.0)
mean PAP, mm Hg	/		40.0 (30.0, 57.0)	38.0 (28.0, 47.0)
mean RAP, mm Hg	/		3.00 (2.00, 5.00)	3.00 (2.00, 4.00)
PVR, WU	/		6.65 (4.25, 11.8)	6.20 (4.35, 9.30)
CI, L·min^− 1^·m^− 2^	/		2.75 (2.30, 3.30)	2.70 (2.40, 3.30)
SvO_2_, %	/		67.0 (60.0, 74.8)	67.0 (60.0, 70.0)
Pericardial effusion, %	/		33 (35.1%)	57 (38.8%)
COPD, %	/		23 (24.5%)	89 (60.5%) ^***^
Coronary artery disease, %	/		5 (5.32%)	9 (6.12%)
Hypertension, %	/		13 (13.8%)	53 (36.1%) ^***^
Type 2 Diabetes, %	/		3 (3.19%)	21 (14.3%) ^*^
Hyperlipidemia, %	/		26 (27.7)	77 (52.4%) ^***^

Values are expressed as mean ± SD or number (percentage), or median (P25, P75). 6MWD, 6-min walk distance; COPD, chronic obstructive pulmonary disease; CI, cardiac index; LVEF, left ventricular ejection fraction; MPV, mean platelet volume; NT-proBNP, N-terminal pro-B-type natriuretic peptide; PAP, pulmonary artery pressure; PAS, pulmonary artery stenosis; PCT, plateletcrit; PDW, platelet distribution width; PH, pulmonary hypertension; PLT, platelet count; PVR, pulmonary vascular resistance; RAP, right atrial pressure; RV, right ventricle; TAPSE, tricuspid annular plane systolic excursion; WHO,World Health Organization. $ *p* < 0.05 for controls compared with PH group, * *p* < 0.05 for PAS compared with non-PAS, ** *p* < 0.01 for PAS compared with non-PAS, *** *p* < 0.001 for PAS compared with non-PAS

### Enrichment score for megakaryocytes and platelets

The quality control metrics of platelet transcriptomic sequencing was summarized in Table S1. The average sequencing depth of all samples was 44.8 ± 11.5 million reads per sample, the mean Q30 was 89.6 ± 2.05%. After alignment, the mean total mapped ratio was 84.6 ± 7.38% with mRNA mapped ratio of 33.2 ± 6.25%.

**Fig. 1:**
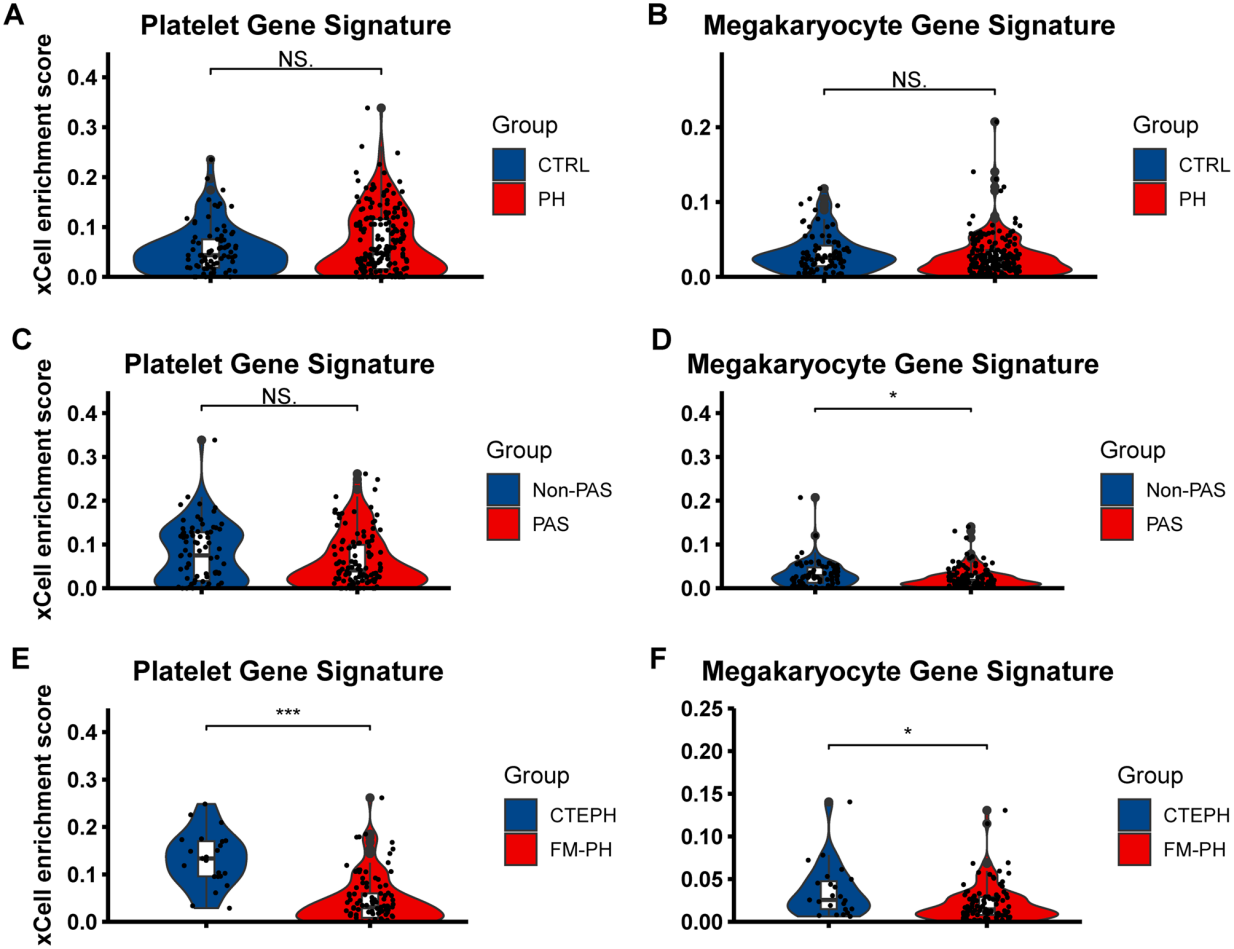
Enrichment score of platelet and megakaryocyte. (**A**-**B**) The level of platelet (**A**) and megakaryocyte (**B**) enrichment score speculated by xCell in PH patients and healthy donors. (**C**-**D**) Platelet (**C**) and megakaryocyte (**D**) enrichment in patients with and without PAS. (**E**-**F**) Platelet (**E**) and megakaryocyte (**F**) enrichment in patients with CTEPH and FM-PH. CTEPH, chronic thromboembolic pulmonary hypertension; FM-PH, PH caused by fibrosing mediastinitis; PAH, pulmonary arterial hypertension; PH, pulmonary hypertension. * *p* < 0.05; *** *p* < 0.001; NS, non-significant

Enrichment scores for platelets and megakaryocytes were subsequently calculated. No significant differences in either enrichment score were observed between the PH and control groups (Fig. 1A-B). The megakaryocyte enrichment score was markedly reduced in PAS patients (Fig. 1C), whereas the difference in platelet enrichment score between the two groups did not reach statistical significance (Fig. 1D). In all PAS samples, CTEPH exhibited a significantly higher enrichment score for both cell types (Fig. 1E-F).

### Identification of DEGs between controls and patients with PH

The batch effects of age in all samples were removed, as indicated by the PCA plot (Fig. S1A). A total of 5741 genes were considered to be expressed, of which 111 were downregulated and 26 were upregulated in the PH group (Fig. S1B). Genes with the highest absolute log_2_fold change were presented (Fig. S1C-D). Subsequent analysis revealed that 26 upregulated DEGs were enriched to KEGG pathways including hypoxia-inducible factor 1 (HIF-1) signaling and focal adhesion (Fig. S1E). GSEA revealed significant upregulation of pathways including mitogen-activated protein kinase and phosphoinositide 3-kinase-protein kinase B (PI3K-Akt) signaling and downregulation of oxidative phosphorylation in platelets from patients with PH (Fig. S1F).

### Development of platelet gene signatures differentiating between PAS and non-PAS in PH patients

**Fig. 2:**
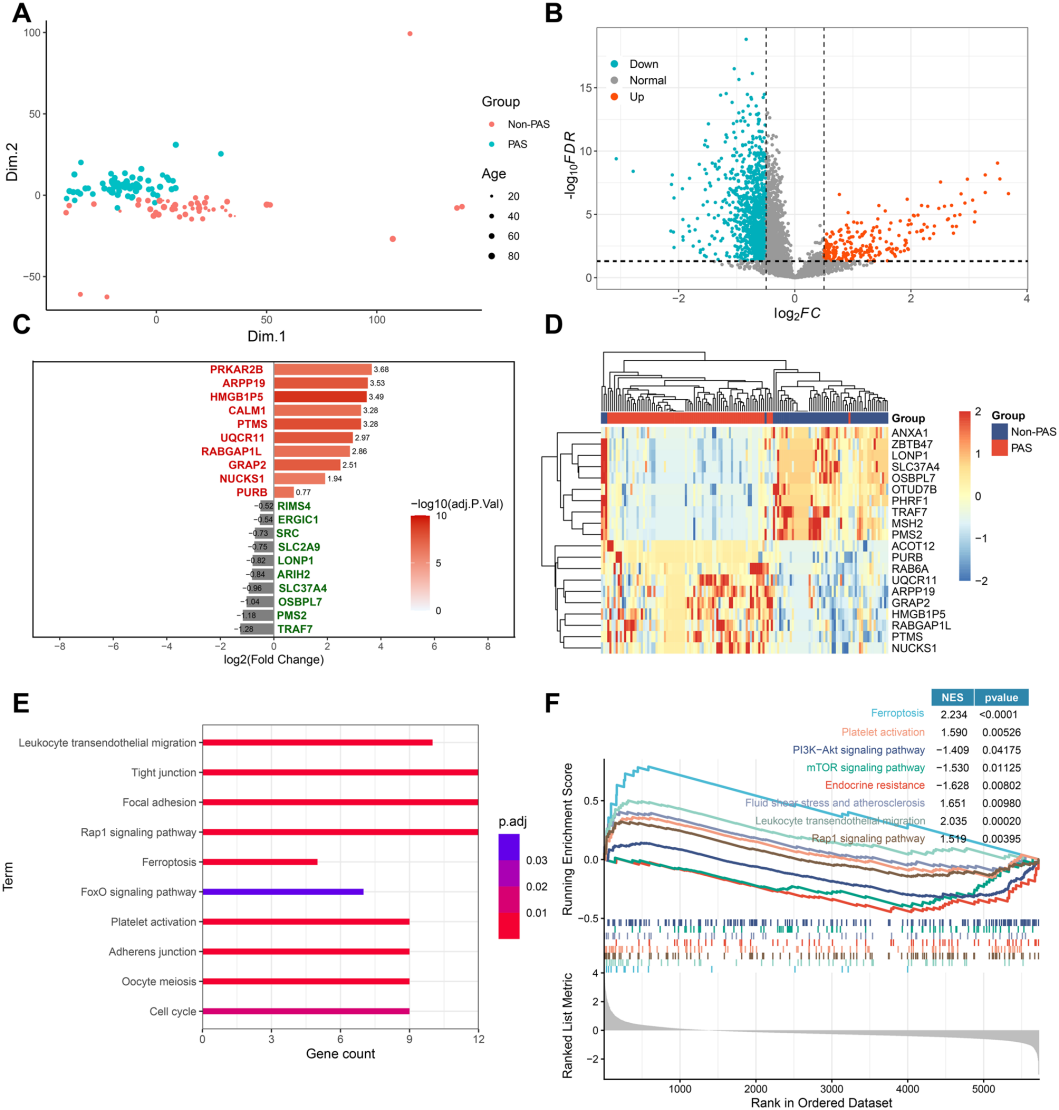
Identification of differentially expressed genes between PH patients with and without PAS. (**A**) PCA plot showing RNA-seq data after removing the batch effect from age. (**B**) Volcano plot showing DEGs between PAS and non-PAS patients. (**C**-**D**) Bar plot (**C**) and heatmap (**D**) showing the top 10 DEGs up- or down-regulated in PAS. (**E**) KEGG gene enrichment analysis of up-regulated DEGs in PAS. (**F**) Results of gene set enrichment analysis utilizing KEGG gene sets, comparing PAS with non-PAS patients. DEGs, differentially expressed genes; PAS, pulmonary artery stenosis; PCA, principal component analysis; PH, pulmonary hypertension; KEGG, Kyoto encyclopedia of genes and genomes

We next compared the platelet transcriptomics between patients with and without PAS and assessed whether platelet RNAs could differentiate PAS from non-PAS using machine learning approaches. The PH samples (*n* = 241) were separated into a training set (*n* = 79 PAS, *n* = 58 non-PAS), an internal validation set (*n* = 35 PAS, *n* = 29 non-PAS), and an external validation set (*n* = 33 PAS, *n* = 7 non-PAS) (Fig. S2). The expression of platelet genes in patients with PH in the training set was analyzed. Following batch effect removal (Fig. 2A), 244 genes were upregulated and 1051 genes were downregulated in the PAS group (Fig. 2B). The top 10 DEGs (Fig. 2C) showed obvious differences in their expression patterns between the two subtypes of PH (Fig. 2D). Functional enrichment analysis indicated that KEGG terms such as platelet activation and Rap1 signaling pathway were enriched in genes upregulated in PAS (Fig. 2E). In comparison to patients devoid of PAS, the PAS cohort demonstrated upregulation of platelet activation, Rap1 signaling pathway, ferroptosis, and fluid shear stress and atherosclerosis, whereas the PI3K-Akt and mechanistic target of rapamycin (mTOR) signaling pathways were significantly downregulated (Fig. 2F).

**Fig. 3:**
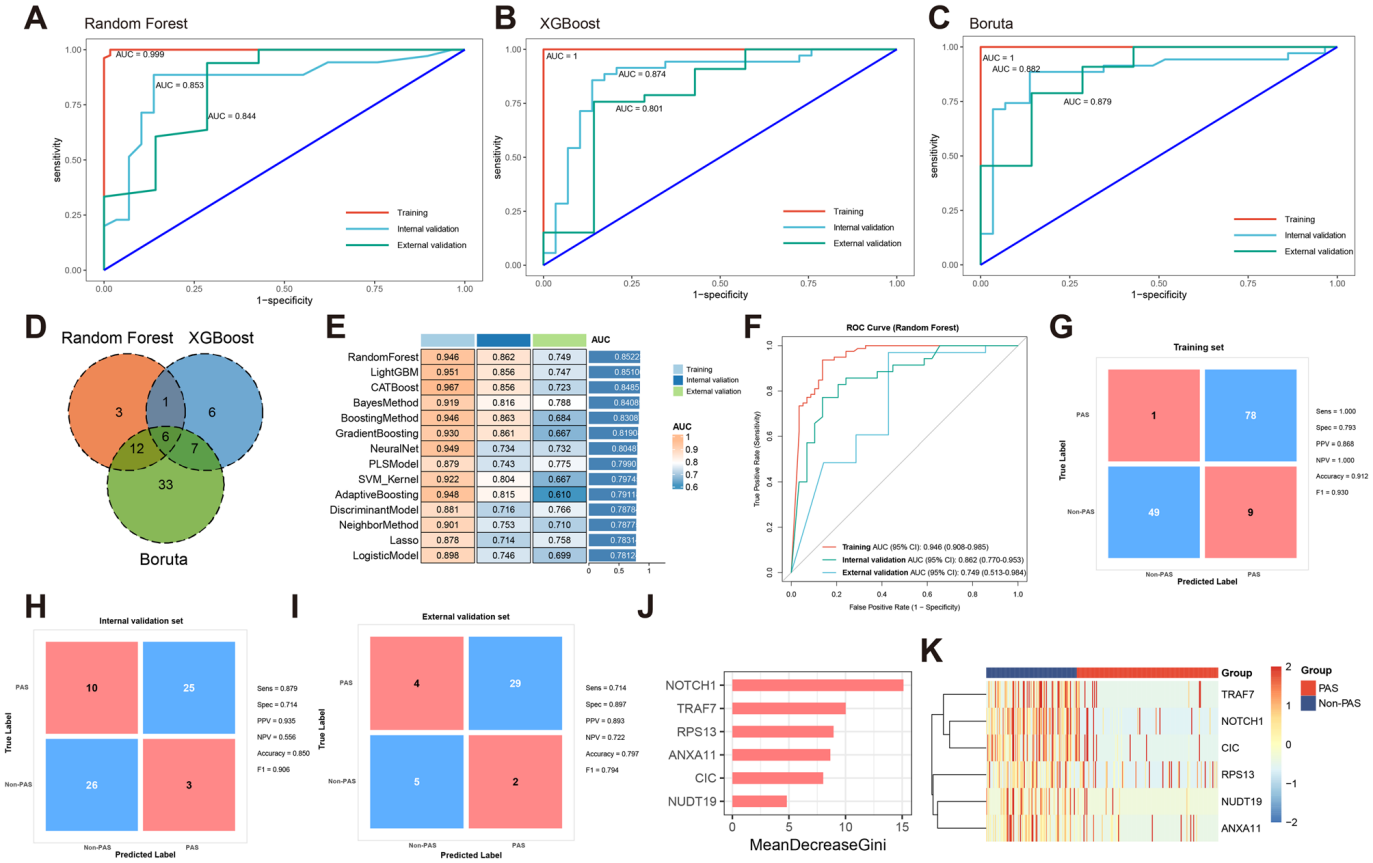
Screening and validation of the platelet RNAs-based model in differentiating PAS from non-PAS PH patients. (**A**-**C**) ROC curves showing the diagnostic performance of RF (**A**), XGBoost (**B**), and Boruta (**C**). (**D**) Venn diagram displaying the intersection of selected genes among the three algorithms. (**E**) Comparison of AUC of 14 machine learning models across training, internal validation, and external validation sets. (**F**) ROC curves showing the diagnostic performance of RF using genes shared by 3 feature selection algorithms. (**G**-**I**) Confusion matrices of RF model in training (**G**), internal validation (**H**), and external validation (**I**) sets. (**J**) In the RF model, variables were ranked by the mean decrease in Gini index. (**K**) Heatmap showing the expression of intersected genes identified by the three feature selection algorithms in patients with PH. AUC, area under curve; RF, random forest; ROC, receiver operating characteristic; PAS, pulmonary artery stenosis; PH, pulmonary hypertension; XGBoost, extreme gradient boosting

To identify the optimal predictors detecting PAS, RF, XGBoost, and Boruta were applied to the training dataset, resulting in 22, 20, and 58 platelet RNAs, respectively (Fig. S3). Each model exhibited favorable discriminative ability, with AUCs of 0.999, 1.000, and 1.000 in the training cohort, respectively (Fig. 3A-C). In the internal validation set, the AUCs of RF, XGBoost, and Boruta models were 0.853, 0.874, and 0.882, respectively, while in the external validation set the AUCs of 3 models remained stable at 0.844, 0.801, and 0.879, demonstrating satisfactory generalizability.

The Venn diagram illustrated the overlap of genes identified by the three algorithms, and 6 genes were jointly selected as robust predictors for subsequent model construction (Fig. 3D). Using these intersected features, 14 machine learning algorithms were trained and compared through repeated fivefold cross-validation. As summarized in the heatmap (Fig. 3E), the RF model achieved the highest average performance across all datasets, with training, internal validation, and external validation AUCs of 0.946, 0.862, and 0.749, respectively. The ROC curves of the RF model further confirmed its strong discriminative capacity (Fig. 3F). Confusion matrices revealed good classification consistency in the training (accuracy = 0.912, F1 = 0.930), internal validation (accuracy = 0.850, F1 = 0.906), and external validation sets (accuracy = 0.797, F1 = 0.794) (Fig. 3G-I). Among 6 platelet RNAs, NOTCH1 showed the highest mean decrease in Gini index, indicating its critical contribution to model prediction (Fig. 3J). The expression of 6 genes in platelets was shown in the heatmap (Fig. 3K), demonstrating downregulation of them in PAS group. These findings suggest that platelet-derived transcripts reflect the molecular heterogeneity of PH and may serve as minimally invasive biomarkers for disease classification.

### Construction of platelet gene signatures distinguishing between CTEPH and FM-PH

**Fig. 4:**
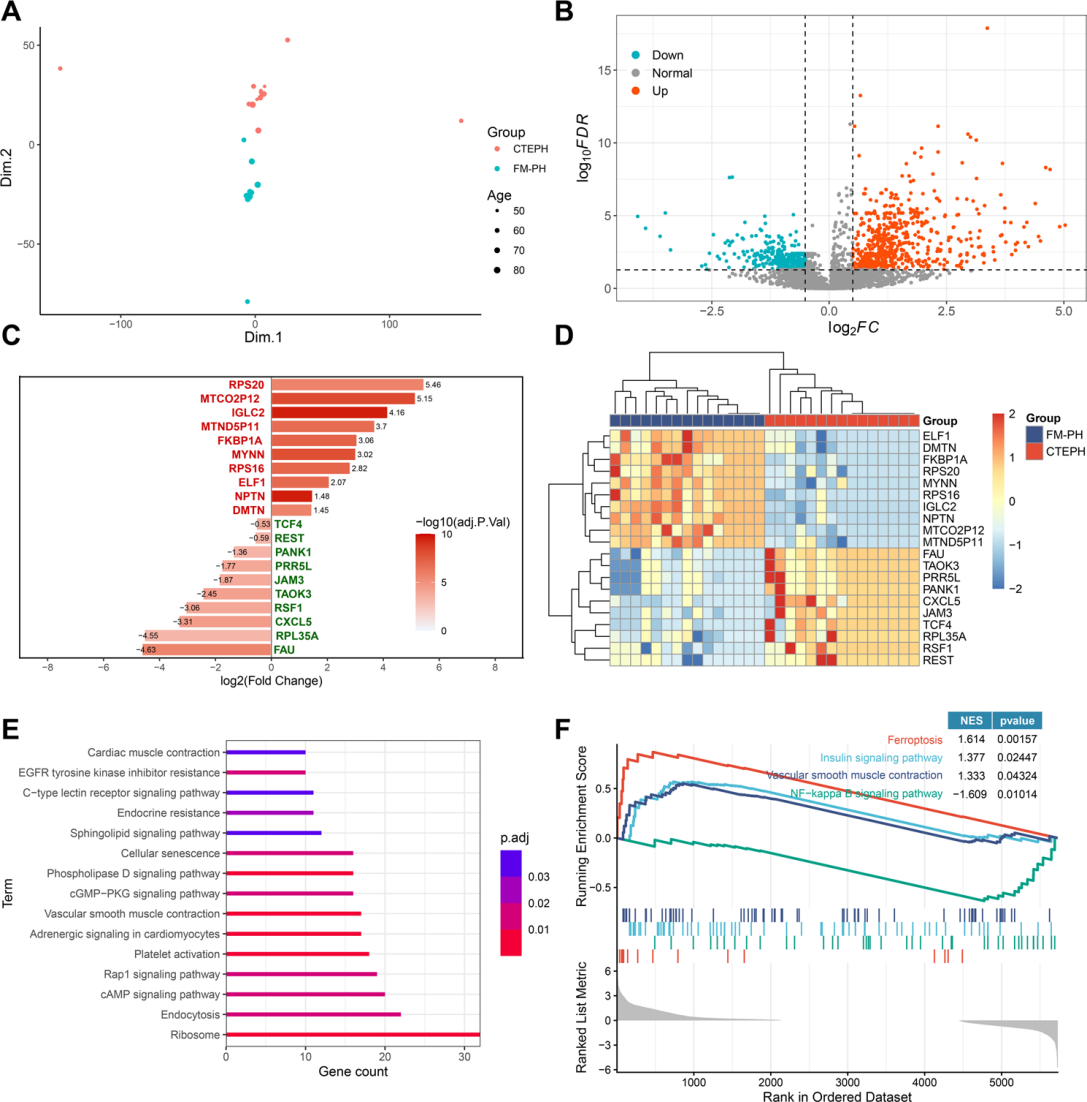
Identification of differentially expressed genes between patients with CTEPH and FM-PH. (**A**) PCA plot showing RNA-seq data after removing the batch effect from age. (**B**) Volcano plot showing DEGs between CTEPH and FM-PH. (**C**-**D**) Bar plot (**C**) and heatmap (**D**) showing the top 10 DEGs up- or down-regulated in CTEPH. (**E**) KEGG gene enrichment analysis of up-regulated DEGs in CTEPH. (**F**) Results of gene set enrichment analysis utilizing KEGG gene sets, comparing CTEPH with FM-PH. CTEPH, chronic thromboembolic pulmonary hypertension; DEGs, differentially expressed genes; FM-PH, PH caused by fibrosing mediastinitis; PCA, principal component analysis; PH, pulmonary hypertension; KEGG, Kyoto encyclopedia of genes and genomes

To identify platelet-derived genes capable of discriminating between CTEPH and FM-PH, we compared platelet transcriptomic profiles from 147 PAS samples. These samples were split into a training set (*n* = 68 FM-PH, *n* = 15 CTEPH), an internal validation set (*n* = 25 FM-PH, *n* = 6 CTEPH), and an external validation set (*n* = 29 FM-PH, *n* = 4 CTEPH). To balance group sizes for DEG analysis in the training set, 15 FM-PH samples were randomly selected. Following elimination of the batch effect (Fig. 4A), 669 genes were upregulated and 697 genes were downregulated in the PAS group (Fig. 4B). The top 10 DEGs (Fig. 4C) showed obvious differences in their expression patterns between the two subtypes of PAS (Fig. 4D). Functional enrichment analysis indicated that pathways of platelet activation, vascular smooth muscle contraction, Rap1 signaling, and cyclic guanosine monophosphate-protein kinase G (cGMP-PKG) signaling were enriched in genes upregulated in CTEPH, compared to FM-PH (Fig. 4E). GSEA indicated that pathways including vascular smooth muscle contraction, insulin signaling and ferroptosis were significantly upregulated in CTEPH (Fig. 4F), whereas nuclear factor kappa-light-chain-enhancer of activated B cell (NF-κB) pathway was significantly downregulated (Fig. 4F). In addition, the pathway of blood vessel remodeling (Fig. S4A) and expression of five out of six genes involved in this set were significantly upregulated in CTEPH (Fig. S4B).

**Fig. 5:**
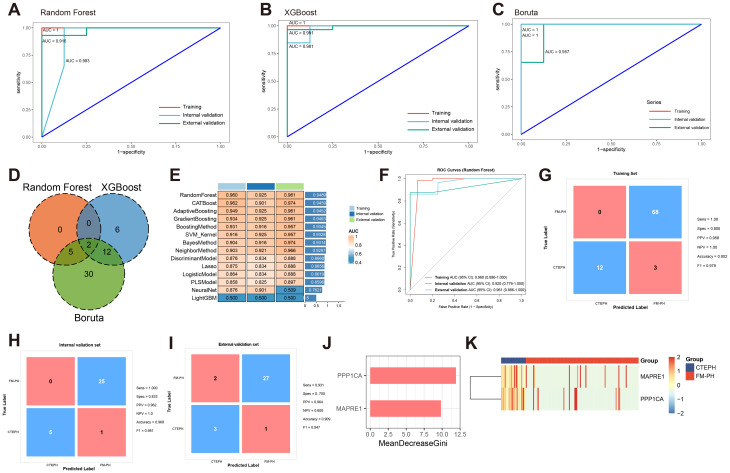
Screening and validation of the platelet RNAs-based model in differentiating CTEPH from FM-PH. (**A**-**C**) ROC curves showing the diagnostic performance of RF (**A**), XGBoost (**B**), and Boruta (**C**). (**D**) Venn diagram displaying the intersection of selected genes among the three algorithms. (**E**) Comparison of AUC of 14 machine learning models across training, internal validation, and external validation sets. (**F**) ROC curves showing the diagnostic performance of RF using genes shared by 3 feature selection algorithms. (**G-I**) Confusion matrices of RF model in training (**G**), internal validation (**H**), and external validation (**I**) sets. (**J**) In the RF model, variables were ranked by the mean decrease in Gini index. (**K**) Heatmap showing the expression of intersected genes identified by the three feature selection algorithms in patients with PAS. AUC, area under curve; CTEPH, chronic thromboembolic pulmonary hypertension; FM-PH, PH caused by fibrosing mediastinitis; RF, random forest; ROC, receiver operating characteristic; PAS, pulmonary artery stenosis; PH, pulmonary hypertension; XGBoost, extreme gradient boosting

Subsequently, RF, XGBoost, and Boruta selected 7, 20, and 49 genes from DEGs between CTEPH and FM-PH (Fig. S5). All three algorithms exhibited excellent discriminative performance in training, internal validation, and external validation sets (Fig. 5A-C). PPP1CA and MAPRE1 were shared by 3 algorithms (Fig. 5D), using which 14 machine learning models were constructed under fivefold cross-validation. As summarized in the heatmap (Fig. 5E), the RF model achieved the best overall performance, with AUCs of 0.960, 0.925, and 0.961 in the training, internal validation, and external validation sets, respectively. ROC curve analysis further demonstrated the superior discriminative capacity of the RF model (Fig. 5F). Confusion matrix analyses revealed consistent predictive performance in all datasets, with high accuracy in the training (accuracy = 0.952, F1 = 0.979), internal validation (accuracy = 0.968, F1 = 0.981), and external validation sets (accuracy = 0.909, F1 = 0.947) (Fig. 5G-I). The feature importance and gene expression of PPP1CA and MAPRE1 were shown in Fig. 5J and K.

## Discussion

The diagnosis of PAS is often overlooked or delayed because of non-specific symptoms such as dyspnea on exertion and fatigue. In addition, according to the current diagnostic flowchart of PH, the diagnosis of PAS might be concealed by comorbidities like left heart disease and chronic pulmonary disease [3]. Previous studies demonstrated the median delays of 14.1-30 months for CTEPH diagnosis [12, 41] and mean delays of 3 years for FM diagnosis [11]. Since the treatment for PH with or without PAS is hugely different and significantly affects patients’ outcomes, the early identification of PAS in patients with PH is of vital importance. Although PAH and Group 3 PH are etiologically distinct and may exhibit differences in platelet biology, both represent forms of PH characterized by the absence of PAS and predominated by remodeling of pulmonary vessels. Importantly, chronic pulmonary diseases are prevalent in patients with CTEPH [42] and FM-PH [43], underscoring the overlapping clinical and pathophysiological features of PH. As a result, Group 3 PH patients were included in the non-PAS cohort, and platelet transcriptomes from PAH and Group 3 PH patients were analyzed together as a representative non-PAS PH group. This strategy enabled comparison with PAS-associated PH to delineate PAS-specific platelet transcriptomic signatures, thereby facilitating discrimination between these two clinically distinct groups of PH.

Utilizing platelet gene signatures selected by 3 feature selection algorithms, RF model achieved an AUC of 0.749 for differentiating PAS from non-PAS and 0.961 for distinguishing CTEPH from FM-PH in independent external validation sets, with sample sizes of 40 and 33, respectively. These results indicated the potential utility of platelet gene signatures in enhancing the assessment of PH etiology and distinguishing FM-PH from CTEPH. Among the platelet signatures, NOTCH1 emerged as the most critical predictor distinguishing PAS from non-PAS. Chaurasia et al. [44] demonstrated that Notch1 signaling within platelets, dependent on PI3K-Akt pathway, was involved in platelet activation, aggregation, and granule secretion, contributing to arterial thrombus formation. Beyond its established role in vascular biology [45], the elevated expression of NOTCH1 observed in the non-PAS group in the present study potentially suggested that Notch1 may participate in thrombosis in situ or vascular remodeling of PAH or Group 3 PH by activating platelet aggregation and release of inflammatory mediators [46]. PPP1CA and MAPRE1, identified consistently across multiple algorithms differentiating CTEPH from FM-PH, are implicated in cytoskeletal and contractile regulation. PPP1CA encodes a catalytic subunit of protein phosphatase 1 that modulates myosin dephosphorylation and vascular tone [47], while MAPRE1 controls microtubule dynamics and fibroblast migration [48]. In this study, both genes were upregulated in platelets from CTEPH patients. Given their roles in regulating cytoskeletal function, these changes may reflect altered mechanotransduction of platelets contributing to thrombosis organization, intimal fibrosis, and vascular remodeling in CTEPH [49]. The elevated expression of PPP1CA and MAPRE1 may further indicate platelets’ adaptive responses to altered mechanical microenvironments of pulmonary arteries, distinguishing CTEPH, characterized by organic thrombotic lesions and vascular remodeling, from FM-PH, where compression of vessels by fibrotic tissue predominates. However, these functional implications remain speculative and require experimental validation in future studies.

Previous studies revealed that patients with CTEPH and PAH exhibited lower platelet counts and elevated MPV than controls [50, 51]. In this study, platelet counts and PCT were significantly higher in patients with PAS than in those without PAS, whereas megakaryocyte enrichment scores were significantly lower in patients with PAS than in those without PAS. Given recent evidence indicating that bone marrow, rather than lung, is the primary site for platelet biogenesis [15], these differences in circulating platelet indices are unlikely to reflect enhanced pulmonary platelet production in PAS. Instead, they may reflect the altered activation or redistribution of platelets in response to focal high shear stress and flow disturbance in PAS, as opposed to diffuse vascular remodeling in non-PAS [21], although further studies are needed to clarify the underlying mechanisms. In addition, the baseline data showed that patients with PAS had significantly lower levels of NT-proBNP and 6MWD. This could be partly explained by the higher proportion of patients with older age and a history of COPD in the PAS group, which could affect patients’ pulmonary function and exercise capacity [52, 53].

In this work, we observed significant upregulation of pathways relevant to platelet activation, Rap1, fluid shear stress and atherosclerosis, and leukocyte transendothelial migration in PAS group. Rap1 was involved in multiple platelet responses including platelet activation [54–56], and could be activated by PI3K-Akt axis in platelets [57]. However, our data indicated that PI3K-Akt signaling was significantly downregulated in PAS. This discrepancy might be explained by a mechanistic hypothesis that pathological fluid shear stress serves as the primary driver of platelet activation under PAS conditions, largely bypassing the canonical Akt-dependent pathways. PH associated with PAS including CTEPH and FM-PH are characterized by chronic vascular narrowing and complex flow disturbances that may expose circulating platelets to elevated fluid shear stress and promote platelet activation [58]. Kawano et al. [59] revealed that shear-induced platelet aggregation was increased in patients with chronic myocardial ischemia, suggesting that platelets might be activated by high fluid shear stress in stenosed coronary arteries. Moreover, Nobili et al. [60] demonstrated in an in vitro model that platelet activation increased in response to dynamic and repeated fluid shear stress exposure with platelet-surface interactions blocked, indicating a fluid shear stress-induced mechanism of platelet activation that operates independently of canonical biochemical signaling pathways. Collectively, these findings suggest that the elevated platelet activation might arise as a consequence of pathophysiological changes in PAS. Nevertheless, further experimental studies are required to validate this proposed hypothesis.

The pathophysiology of CTEPH, while primarily associated with obstruction of blood vessels by organic thrombus [61], also includes small-vessel disease and remodeling of pulmonary arterioles [62]. By contrast, pulmonary arteries in patients with FM-PH are compressed by proliferative fibrous tissue in the mediastinum [4]. In this study, we compared the platelet indices and transcriptomes of these two diseases to identify the mechanistic differences. Compared with FM-PH, platelets from CTEPH patients showed higher platelet and megakaryocyte enrichment scores, and pathways including insulin signaling, vascular smooth muscle contraction, and blood vessel remodeling were upregulated in CTEPH. Considering the potential antiplatelet effect of insulin [63], these results might be explained by a pathophysiological model wherein chronic platelet activation at the site of vascular obstruction drives disease progression. This is supported by studies demonstrating that platelet-derived products can directly stimulate vascular smooth muscle cell migration via inducing formation of microvesicles and matrix metalloproteinase 9 activity [64], and can upregulate the thrombin receptor on these cells via release of transforming growth factor-β1 and platelet-derived growth factor-AB [65], thereby amplifying their responsiveness to mitogenic stimuli. Thus, the platelet transcriptome in CTEPH appears to reflect its primary thrombotic pathology and active role in vascular remodeling.

This study has several limitations. Firstly, some patients participating in this study underwent RHC for follow-up, potentially introducing selection bias due to the treatment of some individuals with PH. Secondly, we did not analyze platelets from patients with PAS such as Takayasu arteritis and congenital PAS at the transcriptomic level, which may limit the representativeness of this study. Finally, the sizes of external validation cohorts were relatively small, which limited the generalizability and robustness of the proposed platelet-derived biomarkers and increase the risk of overfitting. Therefore, the findings of this study should be interpreted cautiously and warrant validation in larger, multi-center cohorts with more diverse clinical characteristics.

## Conclusions

In conclusion, this study examined the transcriptomic features of blood platelets in patients with PH and identified biomarkers capable of detecting PAS in PH patients as well as differentiating CTEPH from FM-PH in patients with PAS. These findings may pave the way for a non-invasive platelet-based diagnostic tool for stratifying PH etiology in clinical settings.

## Supplementary Information

Below is the link to the electronic supplementary material.

Supplementary Material 1

## Data Availability

The data that support the findings of this study are available from the corresponding authors upon reasonable request.

## Abbreviations

PH: Pulmonary hypertension
PAS: Pulmonary artery stenosis
CTEPH: Chronic thromboembolic pulmonary hypertension
FM-PH: Pulmonary hypertension caused by fibrosing mediastinitis
PAH: Pulmonary arterial hypertension
RNA-seq: RNA sequencing
GSEA: Gene set enrichment analysis
KEGG: Kyoto encyclopedia of genes and genomes
DEGs: Differentially expressed genes
RF: Random forest
ROC: Receiver operating characteristic
AUC: Area under the curve
