# Supplementary material for: Platelet gene signatures detecting pulmonary artery stenosis in patients with pulmonary hypertension
Source: Orphanet J Rare Dis. 2026 Mar 13;21:163. doi: 10.1186/s13023-026-04307-4 (PMC13101180; doi:10.1186/s13023-026-04307-4)

**Table S1**. Summary statistics of quality control for platelet transcriptomics data.

| **Quality Control Metric** | **Study Population**  **(N = 333)** |
| --- | --- |
| Clean Q30 (%) | 89.6 (2.05) |
| Total mapped ratio (%) | 84.6 (7.38) |
| mRNA mapped ratio (%) | 33.2 (6.25) |
| Sequencing depth (Reads, Million) | 44.8 (11.5) |

Values are expressed as mean (SD).

**Figure S1**. Identification of differentially expressed genes between pulmonary hypertension and healthy controls. (A) PCA plot showing RNA-seq data after removing the batch effect from age. (B) Volcano plot showing DEGs between PH and controls. (C-D) Bar plot (C) and heatmap (D) showing the top10 DEGs up- or down- regulated in PH. (E) KEGG gene enrichment analysis of up-regulated DEGs in PH. (F) Results of gene set enrichment analysis utilizing KEGG gene sets, comparing PH patients with controls. DEGs, differentially expressed genes; PCA, principal component analysis; PH, pulmonary hypertension; KEGG, Kyoto encyclopedia of genes and genomes.

**
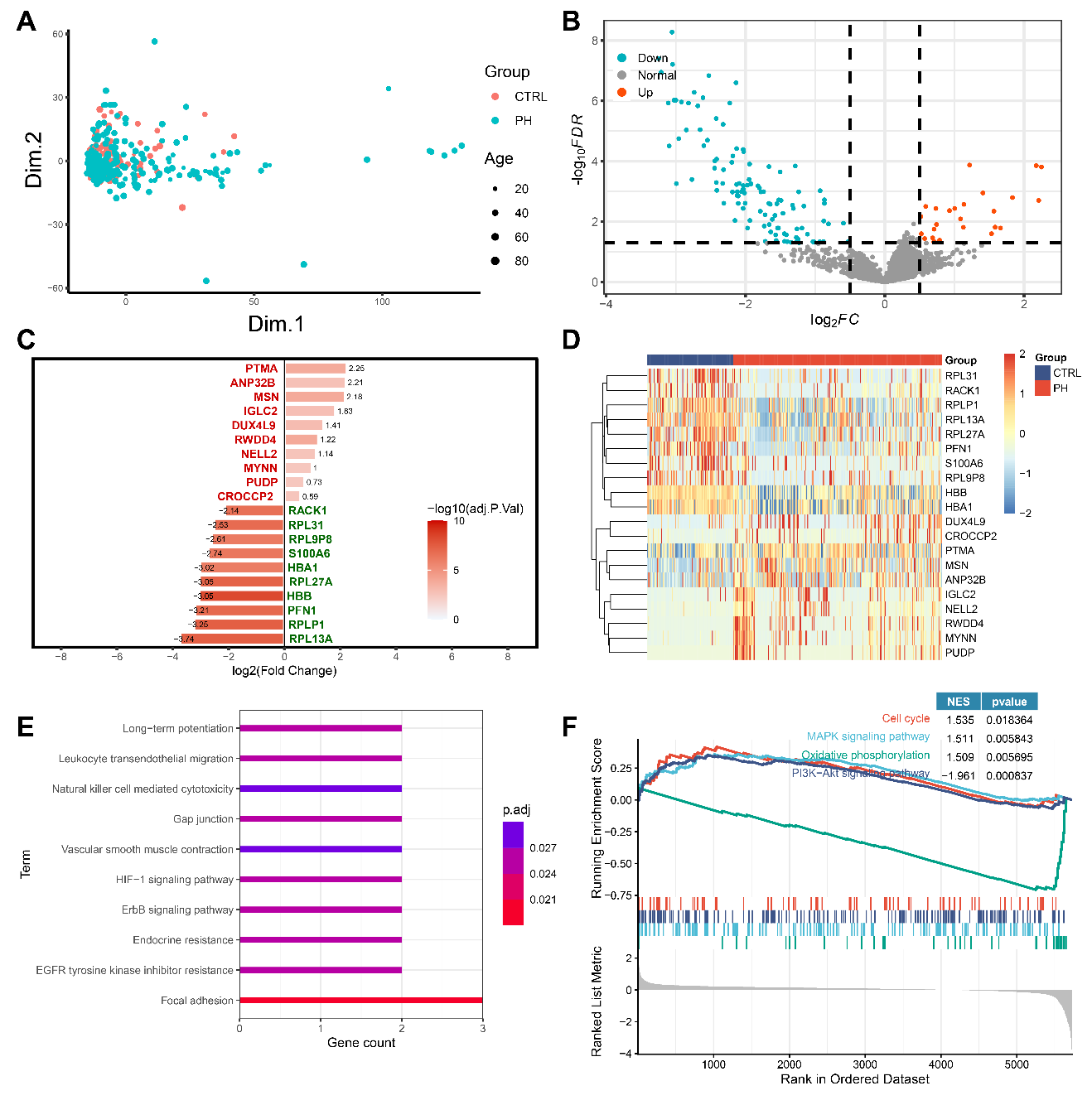
**

**Figure S2**. Series for platelet RNAs-based model development to detect pulmonary artery stenosis. CTEPH, chronic thromboembolic pulmonary hypertension; FM-PH, PH caused by fibrosing mediastinitis; PAH, pulmonary arterial hypertension; PH, pulmonary hypertension.

**
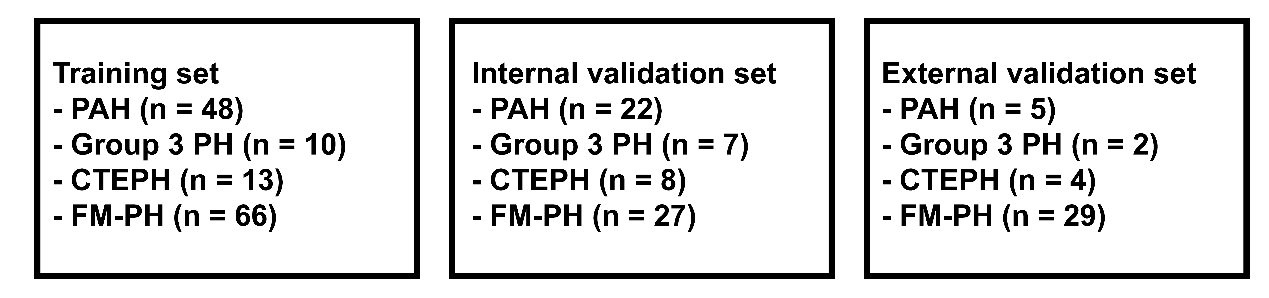
**

**Figure S3**. Selection of platelet RNAs detecting PAS in PH through machine learning algorithms. (A-C) Platelet RNAs selected by RF, XGBoost, and Boruta in terms of differentiating between PAS and non-PAS in patients with PH. PAS, pulmonary artery stenosis; PH, pulmonary hypertension; RF, random forest; XGBoost, extreme gradient boosting.


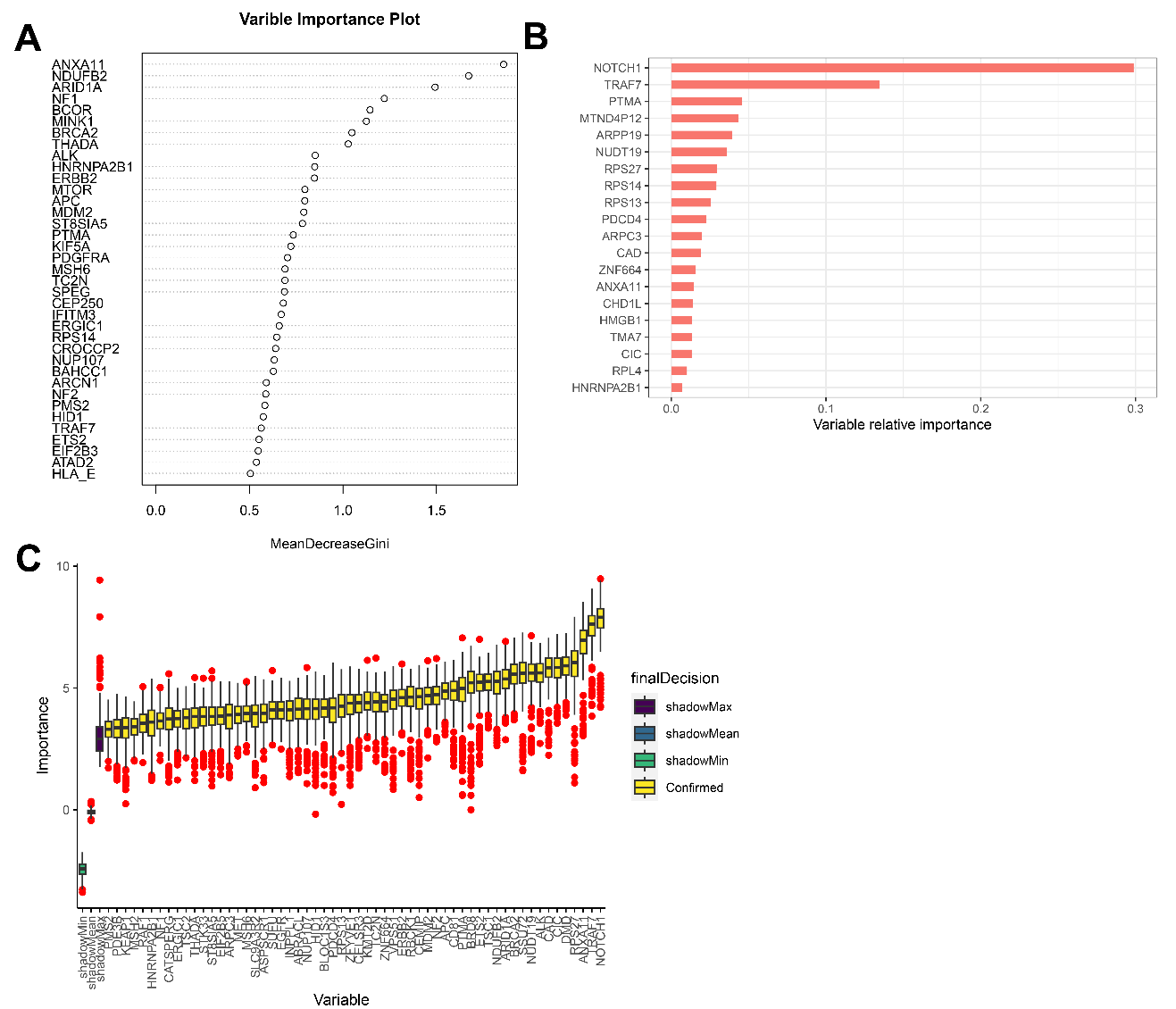


**Figure S4**. Platelet RNAs were associated with blood vessel remodeling in CTEPH. (A) GSEA of blood vessel remodeling pathway comparing CTEPH with FM-PH; (B) Bar plot showing genes involved in the blood vessel remodeling pathway. CTEPH, chronic thromboembolic pulmonary hypertension; GSEA, gene set enrichment analysis. NES,


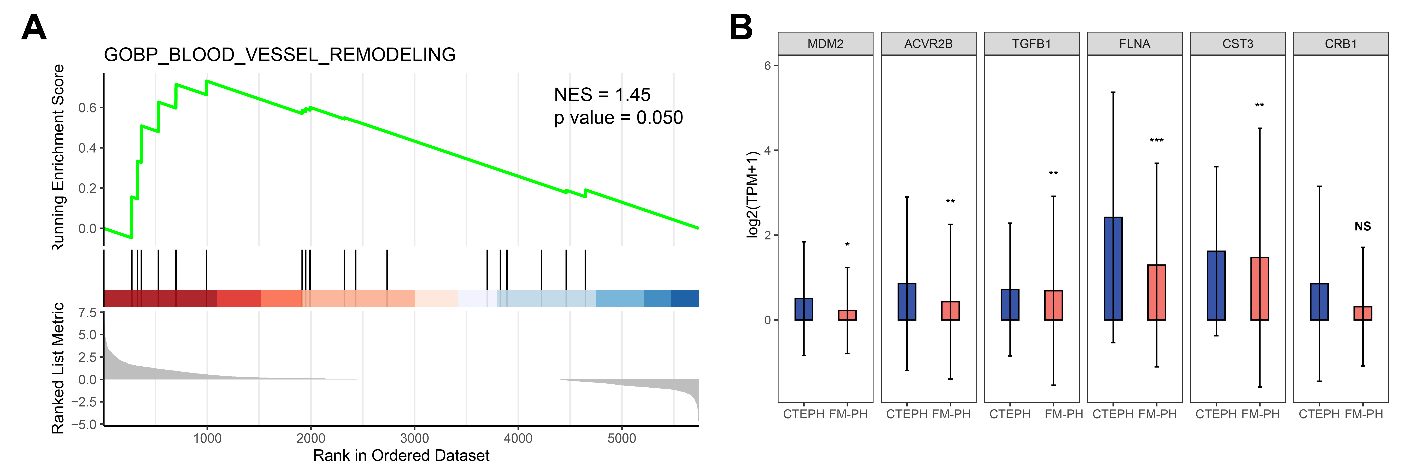


**Figure S5**. Selection of platelet RNAs discriminating CTEPH and FM-PH in PH through machine learning algorithms. (A-C) Platelet RNAs selected by RF (A), XGBoost (B), and Boruta (C) in terms of differentiating between CTEPH and FM-PH. CTEPH, chronic thromboembolic pulmonary hypertension; FM-PH, PH caused by fibrosing mediastinitis; RF, random forest; XGBoost, extreme gradient boosting.


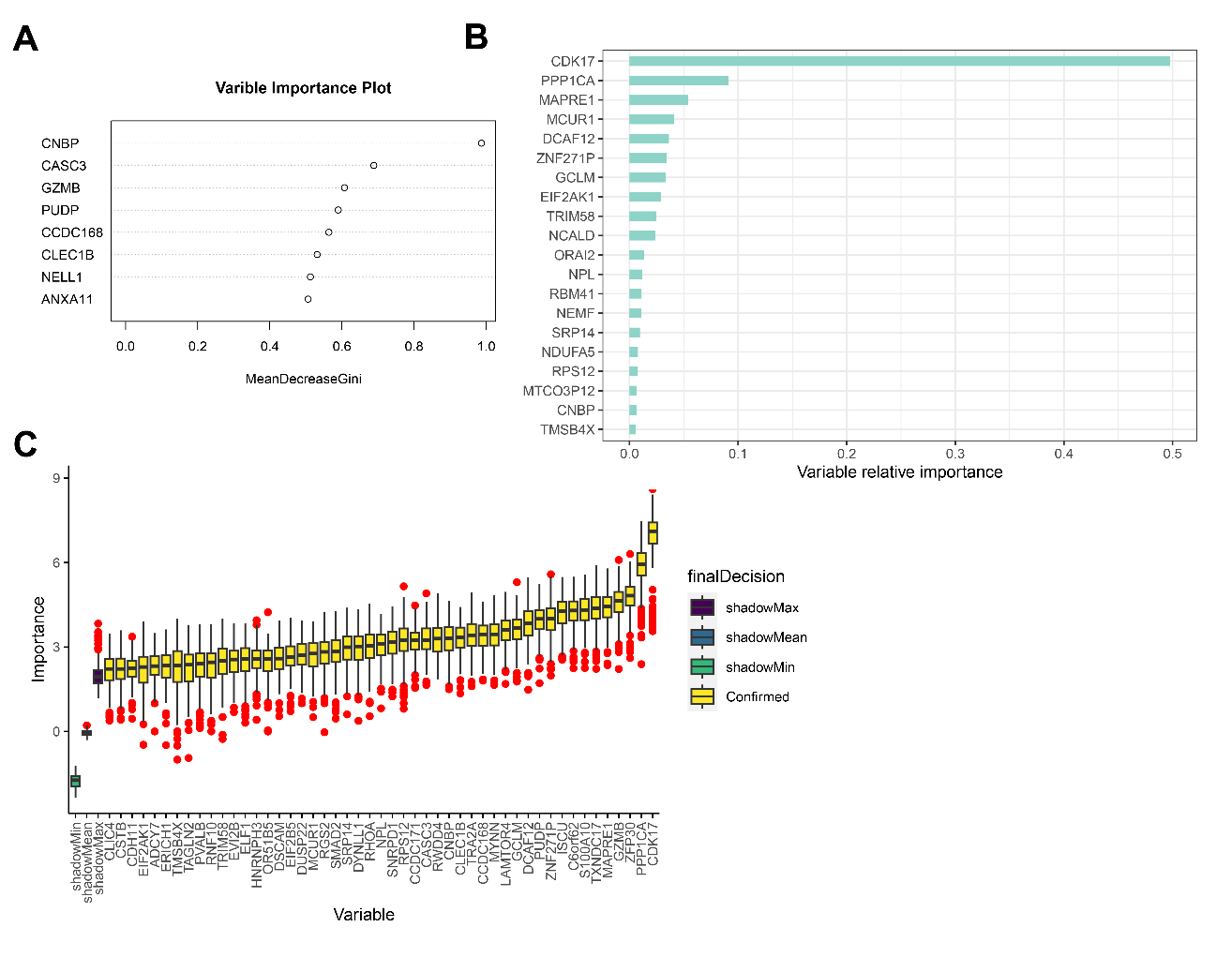

Supplement: Supplementary file 1 — Supplementary Material 1 [file 13023_2026_4307_MOESM1_ESM.docx]
